# Supplementary material for: Antitumor Potential and Phytochemical Profile of Plants from Sardinia (Italy), a Hotspot for Biodiversity in the Mediterranean Basin
Source: Plants (Basel). 2019 Dec 24;9(1):26. doi: 10.3390/plants9010026 (PMC7020228; doi:10.3390/plants9010026)
Supplement: Supplementary file 1 [file plants-09-00026-s001.pdf]

Antitumor potential and phytochemical profile of plants from Sardinia (Italy), a hotspot for biodiversity in Mediterranean basin

Concettina Cappadone[a§], Manuela Mandrone[b§*], Ilaria Chiocchio[b], Cinzia Sanna[c], Emil Malucelli[a], Vincenza Bassi[a], Giovanna Picone[a], Ferruccio Poli[b].

[a] Department of Pharmacy and Biotechnology, University of Bologna, via San Donato 19/2, 40127 Bologna, Italy

[b] Department of Pharmacy and Biotechnology, University of Bologna, Via Irnerio, 42, 40126 Bologna, Italy

[c] Department of Life and Environmental Sciences, University of Cagliari, Via Sant'Ignazio da Laconi 13, 09123, Cagliari, Italy

[§] These authors equally contributed to the manuscript

***Correspondence**

Dr. Manuela Mandrone, University of Bologna, Department of Pharmacy and Biotechnology, Via Irnerio 42, 40126 Bologna, Italy

E-mail: manuela.mandrone2@unibo.it Phone: +390512091294; Fax +39051242576

For special issue 'Bioactive Compounds in Plants'

Table S1. EC_{50} values and respective 95% confidence intervals of the most active plants extracts in U2OS cells, determined by the MTT assay. Data are means of triplicates obtained in one experiment.

Extract	EC_{50} (µM)	
	24h	48h
**AuL**	**125**<br>(85–150)	**95**<br>(87–103)
**CcA**	**96**<br>(88–104**)**	**64**<br>(58–68)
**CycA**	**77**<br>(39–150)	**41**<br>(34–48)
**SaA**	**135**<br>(78–171)	**128**<br>(94–174)
**TaA**	**205**<br>(173–254)	**124**<br>(90–175)

Table S2. Spectral references of the diagnostic signals of the main metabolites detected by ^{1}H NMR profiling in the five most active plants.

Metabolites	Chemical shifts (δ); number and position of protons; multiplicity; coupling constants (Hz)
**alanine**	1.48 (3H-β, d, J = 7.2 Hz)
**β-arbutin**	7.07 (2H-2'-6', d, J = 9.0 Hz); 6.90 (2H-3'-5', d, J = 9.0 Hz); 5.01 (1H-1, d, J = 7.8 Hz)
**caffeic acid**	7.62 (1H-7, d, J = 15.9 Hz); 7.20 (1H-2, d, J = 2.1 Hz); 7.13 (1H-6, dd, J = 8.1, 2.1 Hz); 6.82 (1H-5, d, J = 8.1 Hz); 6.38 (1H-8, d, J = 15.9 Hz)
**cynaropicrin**[a]	6.30 (1H-3'a, d); 6.12 (1H-13a, d); 5.96 (1H-3'b); 5.64 (1H-13b, d); 5.43 (1H-15a, d); 5.32 (1H-15b, d); 5.14 (1-H-14a, d); 4.91 (1-H-14b, d); 4.49 (1H-3, dd); 3.00 (1H-1, ddd); 2.88 (1H-5, ddd); 2.73 (1H-9a, dd); 2.40 (1H-9b, dd,); 2.09 (1H-2a, ddd); 1.74 (1H-2b, ddd)
**formic acid**	8.5 (1H, s)
**α-glucose**	5.20 (1H-1, d, J = 3.8 Hz)
**β-glucose**	4.59 (1H-1, d, J = 7.9 Hz)
**glutamine**	2.82 (2H-γ, m); 2.95 (2H-β, m)
**malic acid**[b]	4.28 (1H-2, dd, J = 6.6, 4.7 Hz); 2.68 (1H3-a, dd, J = 16.6, 4.7 Hz); 2.36 (1H3-b, dd, J = 16.6, 6.6 Hz)
**quinic acid**[b]	1.87 (1H-2a, m); 1.99 (1H-6a, m); 2.01 (1H-2-b, m); 2.08 (1H-6-b, m); 3.40 (1H-4, ov); 4.00 (1H-3, ov); 4.11 (1H-5, ov)

**shikimic acid**	2.19 (1H-6a, dd, J = 18.0, 5.1 Hz); 2.74 (1H-6b, dd, J = 18.0, 5.1 Hz); 3.99 (1H-5, m); 4.40 (1H-3, dd, J = 4.2, 4.2 Hz); 6.45 (1H-2, m)
**succinic acid**[b]	2.44 (4H, s)
**sucrose**	5.40 (1H-1, d, J = 3.8 Hz); 4.17 (1H-3’, d, J = 8.5 Hz)
**trigonellin**	9.14 (1H-2, s); 8.87 (1H-5, m)
**valine**	0.99 (3H-β’, d, J = 6.8 Hz); 1.05 (3H-β’’, d, J = 6.8 Hz)
***O*-rhamnosyl-flavonoid**	0.90 (3H-6’’, d, J = 6.2 Hz)

[a] Complete spectral elucidation of the compound is given in Appendix A.

[b] Changeable by pH and concentration

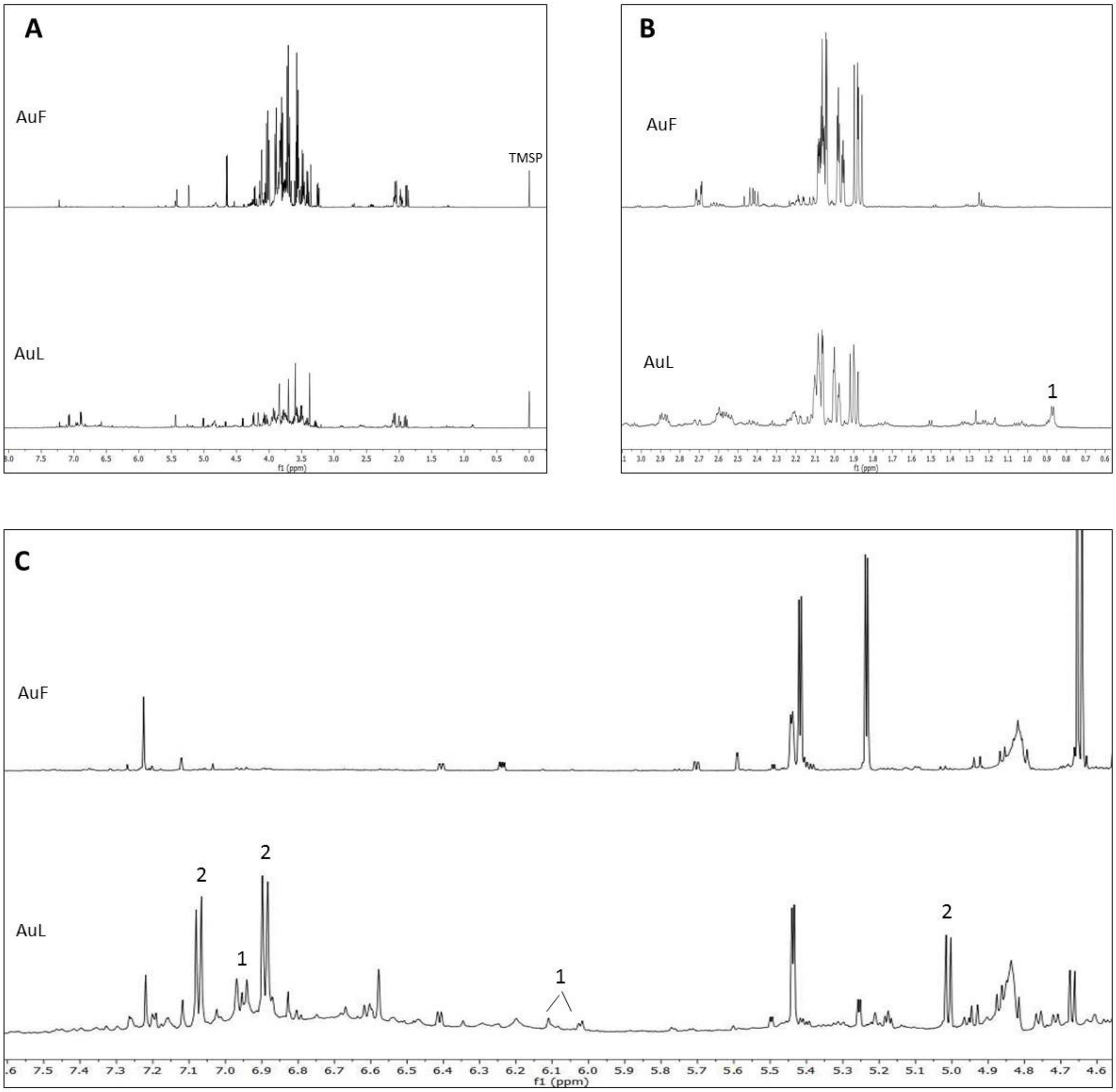

Fig. S1. Comparison between ^{1}H NMR profiles of *Arbutus unedo* fruits (AuF) and leaves extracts (AuL). A) Full spectrum, **B**) extended spectral region from δ 0.8 to 3.0 where is visible the methyl group of rhamnose (1), **C**) extended spectral region from δ 4.6 to 7.5 where are visible the other diagnostic signals of *O*-rhamnosyl flavonoid and signals of arbutin (2). Signals of both compounds are absent in fruits.

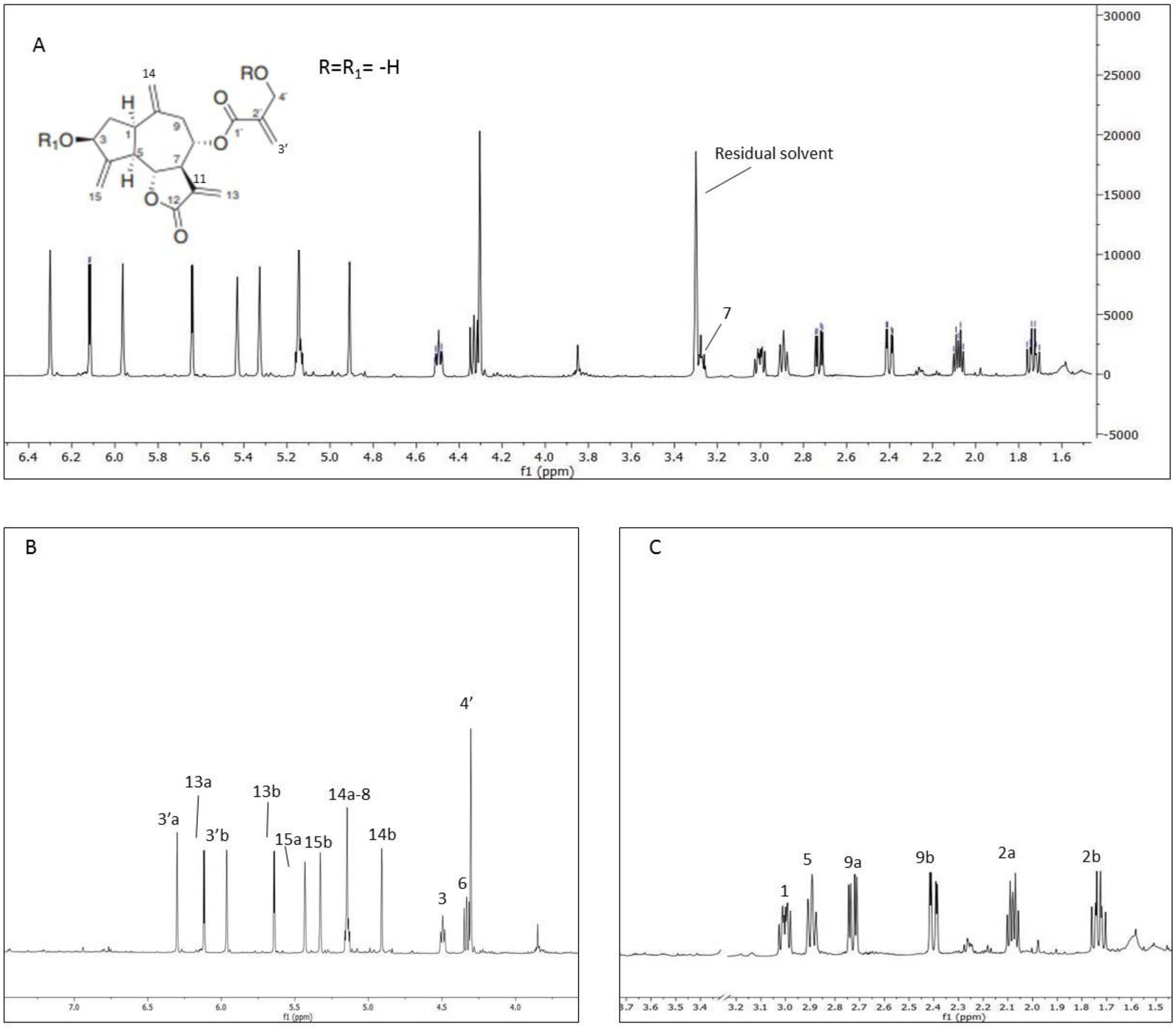

Fig. S2. **^{1}H NMR of CycA chloroform fraction**. **A**) Full spectrum, **B** and **C**) extended spectral regions

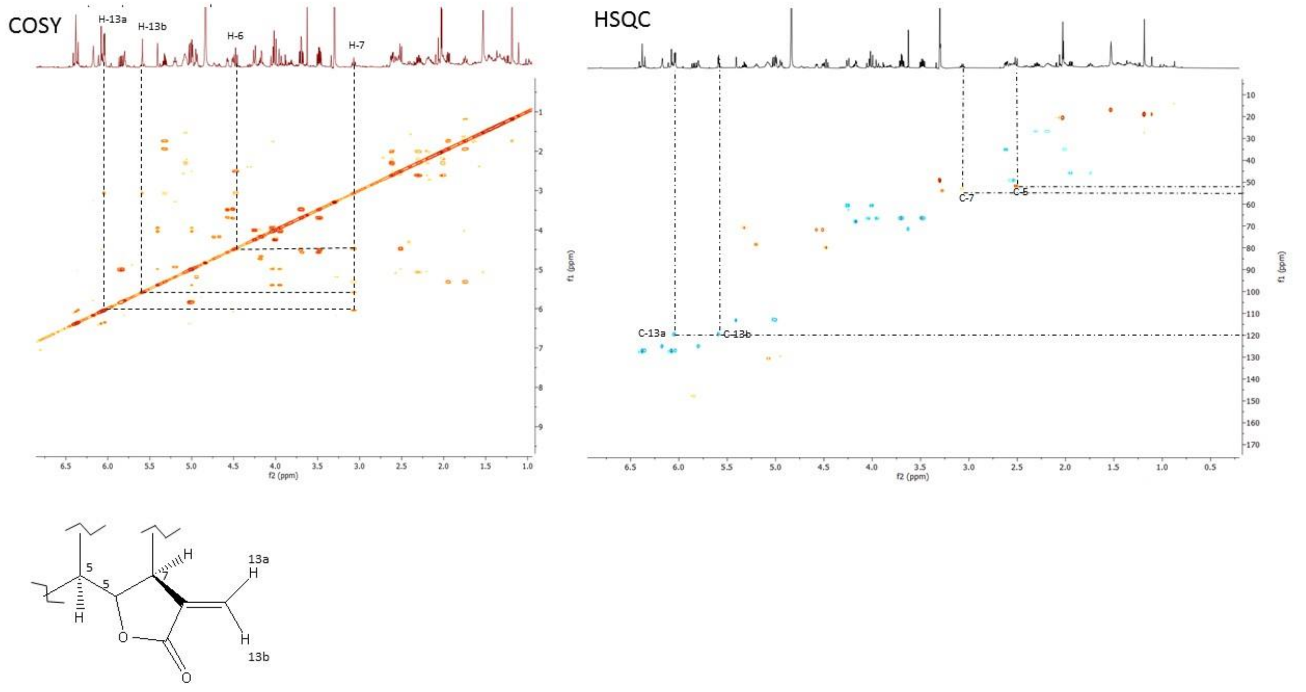

Fig. S3. COSY and HSQC experiment of γ-lactone derivative present in CcA. The correlation highlighted are discussed in the manuscript.

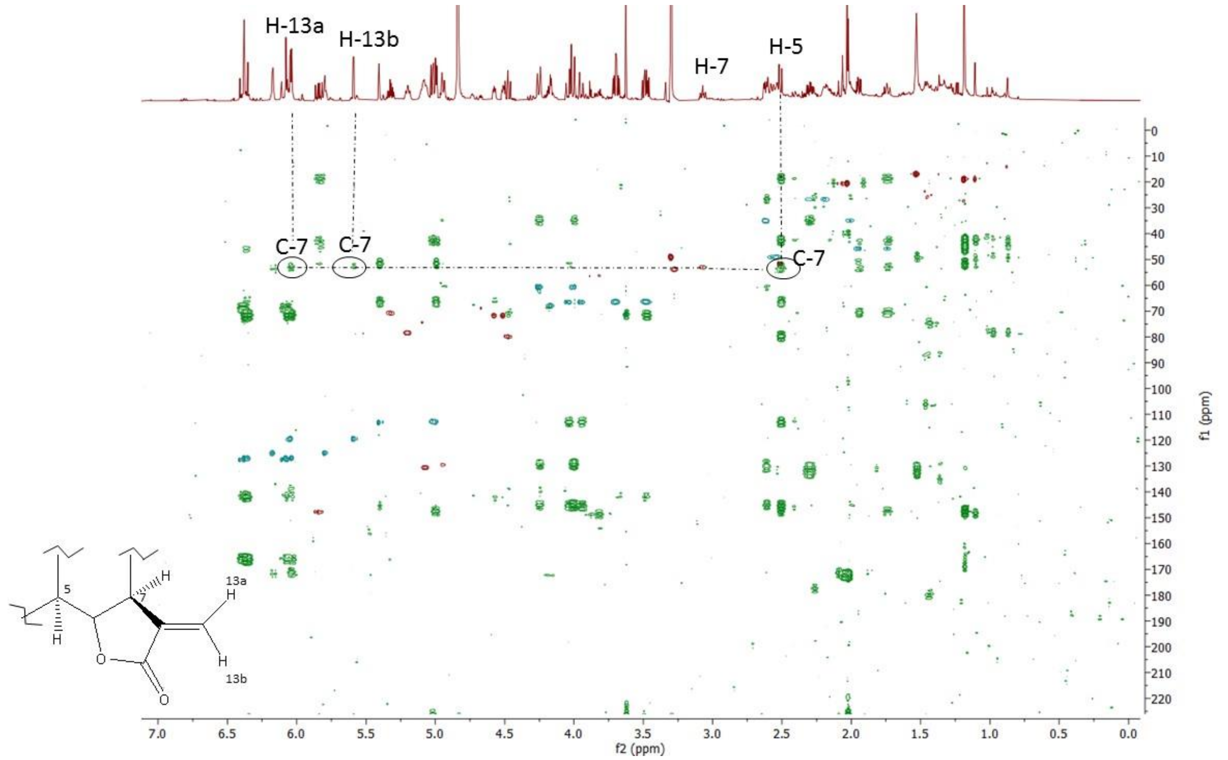

Fig. S4. Superimposed HSQC (red and blue dots) and HMBC (green dots) spectra of γ-lactone derivative present in CcA. The correlation highlighted are discussed in the manuscript.

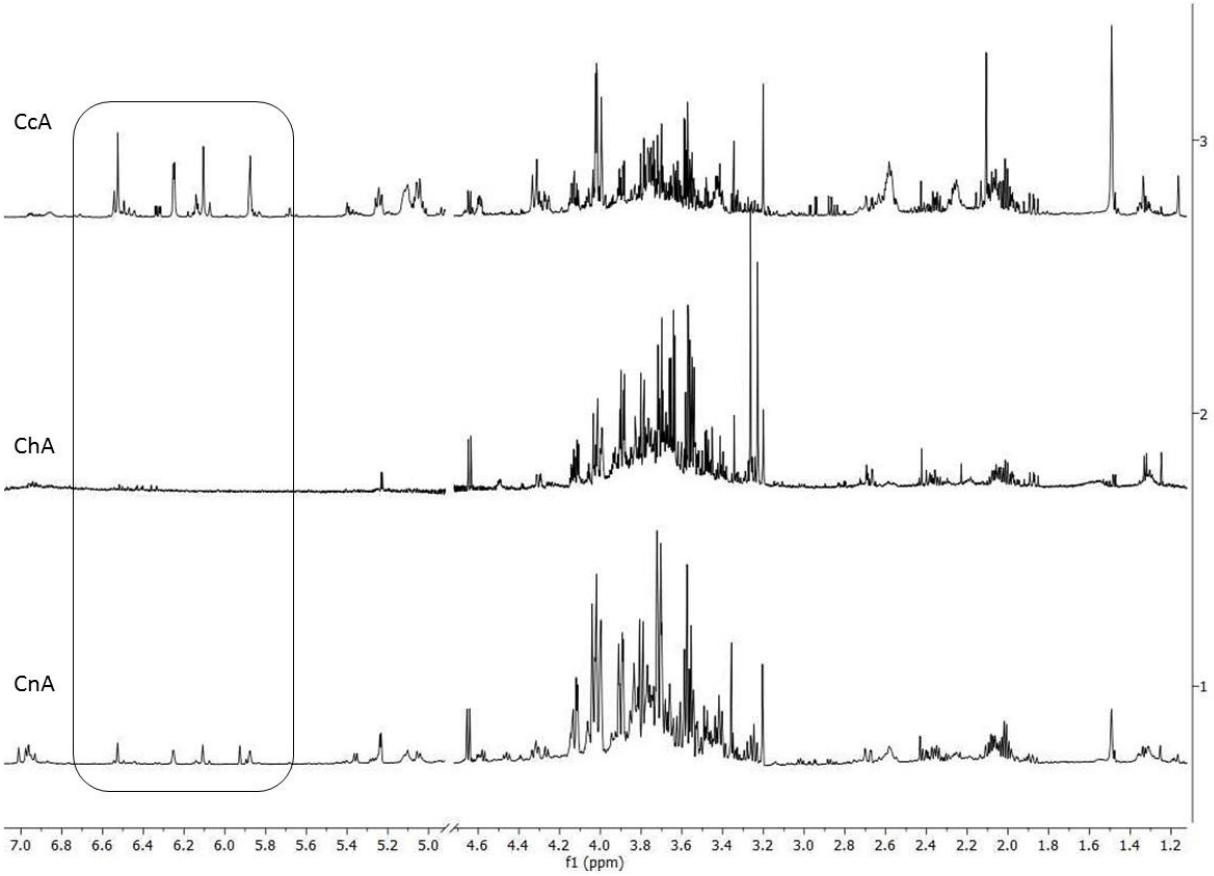

Fig. S5. Comparison among ^{1}H NMR of the three species of *Centaurea, C. calcitrapa* (CcA)*, C. horrida* (ChA)*, C. napifolia* (CnA). Typical signals of lactones sesquiterpenes are highlighted in the square and appear highly concentrated in CcA and scantly or not present in ChA and CnA, which resulted inactive in the bioassays.

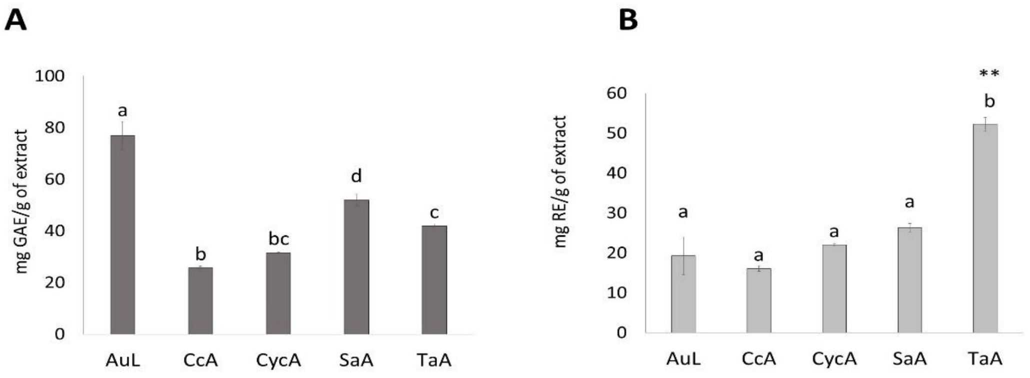

Fig. S6. Total phenolic A) and flavonoid content B) of the five plant extracts. Data are expressed in mg GAE (gallic acid equivalent)/g of extract and of RU eq (rutin equivalent)/g of extract. Means ± S.D. of three experiments analysed in triplicate are reported. The data were analysed by one-way ANOVA and Tukey's post-test. Different letters indicate significant differences at P <0.05, while asterisks (*) indicates P<0.01
